# Upregulation of TCPTP in Macrophages Is Involved in IL-35 Mediated Attenuation of Experimental Colitis

**DOI:** 10.1155/2024/3282679

**Published:** 2024-06-13

**Authors:** Baoren Zhang, Chenglu Sun, Yanglin Zhu, Hong Qin, Dejun Kong, Jingyi Zhang, Bo Shao, Xiang Li, Shaohua Ren, Hongda Wang, Jingpeng Hao, Hao Wang

**Affiliations:** ^1^ Department of General Surgery Tianjin Medical University General Hospital, Tianjin, China; ^2^ Tianjin General Surgery Institute, Tianjin, China; ^3^ School of Medicine Nankai University, Tianjin, China; ^4^ Department of Anorectal Surgery Tianjin Medical University Second Hospital, Tianjin, China; ^5^ Tianjin Key Laboratory of Precise Vascular Reconstruction and Organ Function Repair, Tianjin, China

## Abstract

Ulcerative colitis (UC) is a chronic intestinal inflammatory disease with complex etiology. Interleukin-35 (IL-35), as a cytokine with immunomodulatory function, has been shown to have therapeutic effects on UC, but its mechanism is not yet clear. Therefore, we constructed Pichia pastoris stably expressing IL-35 which enables the cytokines to reach the diseased mucosa, and explored whether upregulation of T-cell protein tyrosine phosphatase (TCPTP) in macrophages is involved in the mechanisms of IL-35-mediated attenuation of UC. After the successful construction of engineered bacteria expressing IL-35, a colitis model was successfully induced by giving BALB/c mice a solution containing 3% dextran sulfate sodium (DSS). Mice were treated with Pichia/IL-35, empty plasmid-transformed Pichia (Pichia/0), or PBS by gavage, respectively. The expression of TCPTP in macrophages (RAW264.7, BMDMs) and intestinal tissues after IL-35 treatment was detected. After administration of Pichia/IL-35, the mice showed significant improvement in weight loss, bloody stools, and shortened colon. Colon pathology also showed that the inflammatory condition of mice in the Pichia/IL-35 treatment group was alleviated. Notably, Pichia/IL-35 treatment not only increases local M2 macrophages but also decreases the expression of inflammatory cytokine IL-6 in the colon. With Pichia/IL-35 treatment, the proportion of M1 macrophages, Th17, and Th1 cells in mouse MLNs were markedly decreased, while Tregs were significantly increased. *In vitro* experiments, IL-35 significantly promoted the expression of TCPTP in macrophages stimulated with LPS. Similarly, the mice in the Pichia/IL-35 group also expressed more TCPTP than that of the untreated group and the Pichia/0 group.

## 1. Introduction

Ulcerative colitis (UC) is a chronic nonspecific inflammatory disease with foci in the colon and rectal tract, and together with Crohn's disease (CD) are known as inflammatory bowel disease (IBD). IBD was initially more prevalent in western countries, but incidence was later found to be increasing in Latin America and Asia [1]. The pathogenesis is complex and ambiguous, for example, genetic [2, 3] and environmental factors [4] lead to weakened intestinal epithelial barrier and imbalanced immune homeostasis. UC usually exhibits symptoms such as abdominal pain, diarrhea, mucopurulent stools, and may also develop concomitant symptoms such as tissue fibrosis, strictures, fistulas, and colon cancer over time [5, 6]. Although hormonal drugs, various immunosuppressive drugs (e.g., azathioprine [7]) and biological agents (e.g., vedolizumab [8]) bring hope to patients, their high cost puts a huge financial pressure on patients, and a large number of patients cannot tolerate. Therefore, some novel therapeutic strategies need to be developed.

IL-35, first identified by Colison et al. [9] in 2007, is an IL-12 family cytokine consisting of a heterodimer of two subunits, Epstein–Barr virus-induced gene3 (EBI3) and IL-12 p35 subunit (IL-12A), which mediates immunosuppressive effects mainly through T cells [9, 10, 11] and B cells [12, 13], and eventually contributes to establishing an immunosuppressive microenvironment. IL-35 not only induces cell cycle arrest in the effector T cell population and inhibits the production of effector cytokines [9, 14] but also induces the proliferation of CD4^+^CD25^+^Foxp3^+^ T cells and the inhibition of T effector cell function [15]. Mice lacking p35 or EBI3 in B cells exhibited aggravated experimental autoimmune encephalitis as well as protection against Salmonella infection, indicating that IL-35 produced by B cells controls overactive immune responses [16]. In addition, dendritic cells transduced with IL-35 gene (IL-35^+^DC) showed no response to TLR stimulation, manifested as failure to increase any costimulatory molecules. Interestingly, IL-12p40 and IL-6 secreted by IL-35^+^DCs were significantly reduced, while IL-10 production was significantly upregulated [17]. However, ongoing studies have demonstrated that IL-35 not only influences T, B, and DC cells but also affects macrophages [18]. Moreover, it has been identified that systemic administration of IL-35 can suppress the activity of UC disease in dextran sulfate sodium (DSS)-induced colitis [19]. Considering the disadvantages of systemic administration [20] such as infection, and vascular toxicity, and the advantages of oral administration, such as minimal side effects and convenient procedure [21], developing a vector continuous expressing IL-35 *in vivo* is worth exploring.

Pichia pastoris (*P. pastoris*) is the most popular host in eukaryotic expression systems for low cost and stable expression. It cannot only perform posttranslational modifications but also secrete recombinant proteins directly into the culture supernatant [22]. Previous studies have demonstrated that various recombinant Pichia pastoris could secrete active protein/cytokine and exhibit positive function [23, 24]. Importantly, Pichia pastoris has been approved as an effective vector for biopharmaceutical use [25], which shows its safety and clinical application prospects. All these provide a solid foundation for the study of whether *P. pastoris* expressing IL-35 (Pichia/IL-35) could improve UC.

The subepithelial layer of the intestinal mucosa is where the immune response occurs and exists many immune cells, with macrophages accounting for a large proportion [26]. Functional changes in macrophages are essential for intestinal homeostasis [27, 28]. The inflammatory response initialed by hyperactivation of macrophages, such as the release of large amounts of cytokines and chemokines, is the dominant cause in the pathogenesis and continuous progression of UC [29]. T cell protein tyrosine phosphatase (TCPTP), also known as protein tyrosine phosphatase nonreceptor 2 (PTPN2), is an intracellular protein tyrosine phosphatase (PTP) that is widely expressed in various cells (such as, intestinal epithelial cells, macrophages, and T lymphocytes) and regulates immune responses by dephosphorylating target proteins [30, 31, 32, 33]. Mutations of gene encoding TCPTP result in various autoimmune diseases such as IBD [34] and rheumatoid arthritis [35]. *In vitro* and *in vivo* experiments have proved that TCPTP can regulate multiple signaling pathways [36, 37, 38, 39] by inhibiting Janus-activated kinases (JAK)/signal transducer and activator of transcription (STAT) [30, 35, 40, 41, 42, 43]. The direct substrates include JAK3, STAT1, and STAT5 [44, 45, 46]. Studies have demonstrated that dephosphorylation of c-jun N-terminal kinase (JNK) in macrophages is a way in which TCPTP prevents inflammatory responses [47]. In addition, Spalinger et al. [48] reported that deletion of TCPTP in macrophages impairs the interaction of macrophages with intestinal epithelial cells and results in the secretion of more inflammatory cytokines. These studies suggest that TCPTP may be a potential downstream effector for IL-35 to alleviate macrophage-related diseases.

Therefore, in this study, we successfully constructed a genetically engineered bacterium expressing IL-35, and investigated their therapeutic effects and underlying mechanisms in the treatment of experimental colitis.

## 2. Materials and Methods

### 2.1. Culture of Pichia/IL-35

IL-35 expressing strains and empty-load strains were purchased from *Gene Universal*. We named them Pichia/IL-35 and Pichia/0, respectively. They were inoculated proportionally in BMGY medium and incubated at 28.5°C until OD_600_ = 2–6. After collecting into centrifuge tubes, replace with BMMY medium (1% methanol) and continue to incubate at 28.5°C for 70 hr. The cells were then collected at 4°C, 12,000 rpm, and 5 min, the expression of IL-35 by anti-IL-12A antibody (Abcam, UK) and anti-His tag antibody (Solarbio, Beijing, China) was detected by western blotting.

### 2.2. Animals

The entire experiment followed the guiding protocol of the China Animal Protection Committee and was approved by the Animal Ethical and Welfare Committee of Tianjin Medical University General Hospital (IRB2022-DW-25). The SPF BALB/c male mice of 6–8 weeks and 22–25 g were purchased from the State Food and Drug Administration of China and housed in a standard environment at the Tianjin Institute of General Surgery, where they had free access to food and water and could adapt to light conditions and ambient temperature before the experiments.

### 2.3. Experimental Groups

The mice were randomly divided into three groups: DSS group, DSS + Pichia/0 group, and DSS + Pichia/IL-35 group, with six mice in each group. Colitis was induced in mice according to the method described previously. Briefly, mice were fed with distilled water containing 3% DSS (YEASEN, China) for 5 days, and then replaced with distilled water for 5 days [20]. During the 5-day period of replacement with normal distilled water, mice in the DSS group were given 0.2 mL of PBS per day via the orogastric tube. In the DSS + Pichia/IL-35 group, 0.2 mL PBS suspensions (1 × 10^10^ CFU/mouse/day) of Pichia/IL-35 induced with methanol was given to the mice and an equal amount of PBS containing Pichia/0 was given to the DSS + Pichia/0 group. On the 10th day after modeling, the mice were all executed with cervical dislocation, leaving the spleen and intestine for subsequent experiments. Body weight changes, blood in stool, and stool consistency were recorded daily for each mouse in order to calculate the disease activity index (DAI) based on standard criteria [49].

### 2.4. Pathological Examination

The colon samples were washed with PBS and then cut into 5 *μ*m sections after formalin fixation and paraffin embedding steps. The sections were stained with hematoxylin–eosin after dewaxing and hydration steps and then scored for inflammation according to the previously published scoring criteria [50, 51].

### 2.5. TUNEL Assay

After dewaxing the slides with the dewaxing solution, each group of samples was tested for apoptosis according to the kit method (Elabscience, China). After coverslips were covered, images were collected using fluorescence microscope. The data were finally processed with Image J.

### 2.6. Immunohistochemistry Staining

The expression of CD206, IL-6 in the inflammatory intestine was determined by immunohistochemical staining. Sections were hydrated and then soaked in EDTA solution at 100°C for 15 min to achieve antigen repair. Then, 3% hydrogen peroxide was added dropwise to the sections to eliminate endogenous peroxidase, and 30 min later nonspecific antibodies were blocked again with 10% goat serum, and finally then incubated overnight at 4°C with antibodies against CD206 (1 : 250, Santa Cruz Biotechnology), IL-6 (1 : 800, Abcam, ab6672). After resuming room temperature on the next day, the sections were incubated in reaction promoter, antirabbit IgG antibody (Zhongshan Jinqiao, PV-9000, China) for 20 min at room temperature, respectively. Finally, sections were incubated with freshly prepared DAB solution (Zhongshan Jinqiao, ZLI-9018, China) after washing, and hematoxylin was added dropwise for nuclear staining and then scanned for image analysis. Image analysis was performed using Image J software.

### 2.7. Flow Cytometry Analysis

According to the previous study [20, 52], flow cytometry was used to determine the immune cell population in the mesenteric lymph nodes (MLNs) and macrophages *in vitro*. The MLNs were removed by opening the peritoneal cavity of the mice and made into a single-cell suspension after grinding and centrifugation. Purchase all flow cytometry monoclonal antibodies and reagents used in this experiment from Biolegend or eBioscience, mainly including Zombie NIR™ Dye (Dead/Live reagent), antimouse CD4 (FITC-labelled), IFN-*γ* (PE-labelled), IL-17A (Percp-cy5.5-labelled), CD25 (Percp-cy5.5-labelled), Foxp3 (APC-labelled), CD11b (FITC-labelled), F4/80 (APC-labelled), and CD86 (Percp-labelled, PE-labelled) to detect Th1 (CD4^+^IFN-*γ*^+^), Th17 (CD4^+^IL-17 A^+^), Tregs (CD4^+^CD25^+^Foxp3^+^), and M1 macrophages (CD11b^+^F4/80^+^CD86^+^).

### 2.8. *In Vitro* Cell Culture and Transfection

Bone marrow-derived macrophages (BMDMs) were isolated from femurs and tibias of BALB/c mice. Mice were disinfected by immersion in 75% alcohol after cervical dislocation. Soak the removed femur and tibia in a 10 cm dish with PBS, remove the impurities from the surface of the bone, and then repeatedly rinse the bone marrow cavity with a 1 mL syringe several times until the rinsed liquid becomes clear. After collecting the liquid, centrifuge (2,000 rpm, 10 min) and discard the supernatant. After lysis of red blood cells and centrifugation, the cells were resuspended with a complete medium containing 100 ng/mL M-CSF (DMEM-High Glucose, 10% FBS, P/S) and incubated at 37°C in a 5% CO_2_ incubator. Half volume change on the third day, cells were used for subsequent experiments after complete medium change on the seventh day. The murine macrophage cell line, RAW264.7, was cultured in DMEM-high glucose medium with 10% FBS and 1% P/S at 37°C in a 5% CO_2_ incubator. One hundred nanograms per milliliter of LPS was used to stimulate both types of macrophages and 40 ng/mL was the therapeutic concentration of IL-35. Based on the complete genome of TCPTP, the small interfering RNAs (SiRNAs) were synthesized by Synbio Technologies. Negative control siRNA was also supplied by Synbio Technologies. Transfections were performed by using Lipo 2000 (Sigma–Aldrich, USA) according to the manufacturer's protocol. The normal macrophages and the cells transfected with control SiRNA were used as controls respectively.

### 2.9. Western Blot

*In vitro* cultured cells and colonic tissue homogenates were lysed by RIPA and mixed with PMSF to extract total protein, respectively. Then, proteins from each sample were subjected to 10% sodium dodecyl sulfate-polyacrylamide gel electrophoresis (SDS-PAGE, Solarbio, Beijing, China). After incubation with anti-TCPTP antibody, anti-STAT1 antibody, anti-p-STAT1 antibody, and anti-GAPDH antibody overnight at 4°C, the membranes with blotted proteins were then incubated with HRP-conjugated goat antirabbit secondary antibody (dilution at 1 : 2,000, CST, Boston, USA) or rat antimouse secondary antibody (dilution at 1 : 2,000, Servicebio, Wuhan, China) for 1 hr at room temperature. After washing three times with TBST, a prepared electrochemiluminescent solution (ECL, Millipore, MA, USA) was added dropwise to the membrane and the membrane was then exposed to the exposure machine (ChemiScope series, Clinx Scientific Instruments Ltd.).

### 2.10. Statistics

Data shown in this study were expressed as mean ± SD, and the differences among multiple groups were analyzed using one-way analysis of variance (ANOVA), GraphPad Prism 8 software was applied. Throughout the text, figures, and legends, the following terminologies are used to denote statistical significance: ns = no significant;  ^*∗*^*p* < 0.05;  ^*∗∗*^*p* < 0.01;  ^*∗∗∗*^*p* < 0.001; and  ^*∗∗∗∗*^*p* < 0.0001.

## 3. Results

### 3.1. Identification of Pichia/IL-35

**Figure 1:**
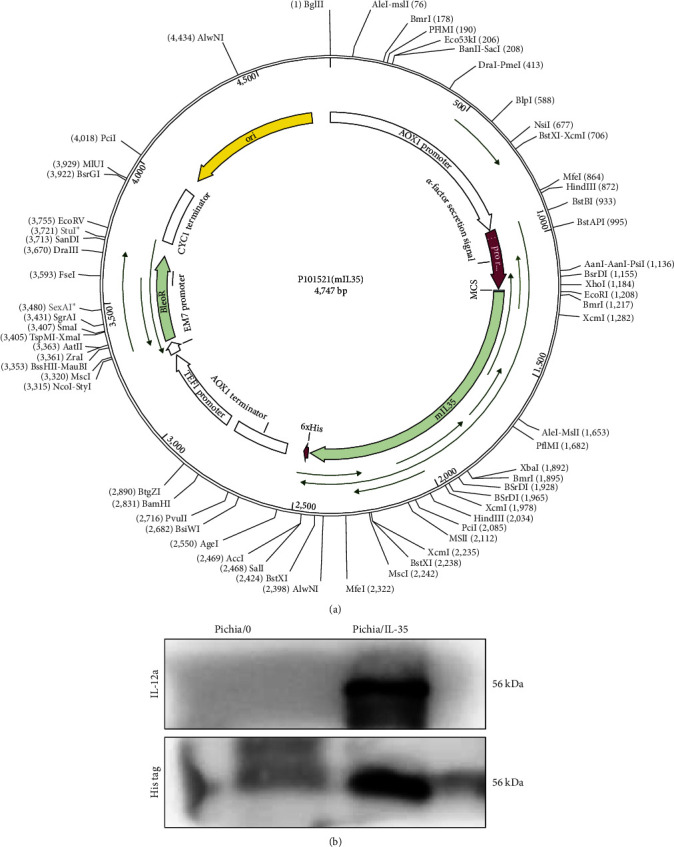
Identification of the successful construction of Pichia/IL-35. (a) Plasmid construction diagram. (b) To ensure the successful construction of Pichia/IL-35, the total cell lysate proteins from the strain Pichia/0 or Pichia/IL-35 were obtained, and a western blot analysis was performed.

Plasmid construction is shown in Figure 1(a). After induction, the expression of IL-35 was detected by western blot. As shown in Figure 1(b), the band of IL-12A and His tag at 56 KDa can be detected in the Pichia/IL-35 group while not be detected in the Pichia/0 group, which identified that the Pichia/IL-35 could stably express IL-35.

### 3.2. Pichia/IL-35 Ameliorated Symptoms of Experimental Colitis

**Figure 2:**
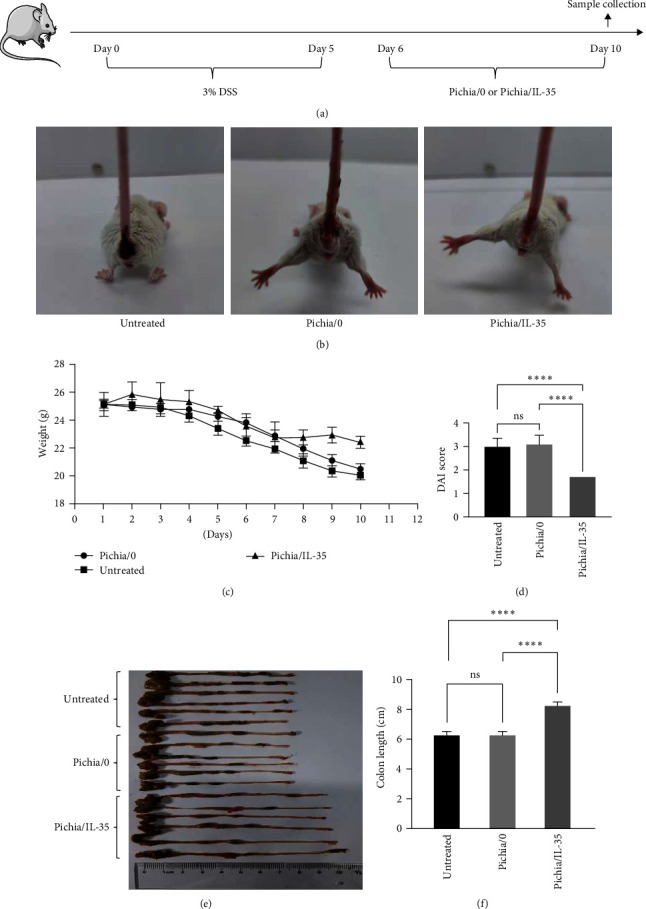
Pichia/IL-35 ameliorated symptoms of experimental colitis. (a) Details of the dextran sodium sulfate (DSS) used for the induction of experimental colitis and treatment of Pichia/0 or Pichia/IL-35. (b) Representative photos showing bloody stool at day 10 after DSS administration. Body weight loss (c) and disease activity index (DAI) score (d) of mice from untreated, Pichia/0 and Pichia/IL-35 groups (*n* = 6 per group). (e) Representative image of the colon after DSS administration and Pichia/0 or Pichia/IL-35 treatment. (f) Colon length of mice from untreated, Pichia/0 and Pichia/IL-35 groups was measured. All data are presented as mean ± SD. One-way ANOVA was used to perform statistical analysis. ns = no significant,  ^*∗∗∗∗*^*p* < 0.0001.

BALB/c mouse colitis was successfully induced by drinking distilled water containing 3% DSS (Figure 2(a)) which was characterized by bloody diarrhea and weight loss, and the DAI score was used to assess the severity of colitis. As shown in Figures 2(b) and 2(c), Pichia/IL-35 treatment could ameliorate bloody stool and weight loss. DAI score in Pichia/IL-35-treated group was significantly lower than that of other groups (Pichia/IL-35 group vs. Pichia/0 group, *p* < 0.0001; Pichia/IL-35 group vs. untreated group, *p* < 0.0001), while colon length in Pichia/IL-35-treated group was much longer than that of other groups (Pichia/IL-35 group vs. Pichia/0 group, *p* < 0.0001; Pichia/IL-35 group vs. untreated group, *p* < 0.0001) (Figures 2(d) and 2(f)). In contrast, results showed that Pichia/0 could not have therapeutic effects on attenuation of colitis (DAI score and colon length: Pichia/0 group vs. untreated group, ns).

### 3.3. Pichia/IL-35 Alleviated Colon Damage and Apoptosis in Colitis

**Figure 3:**
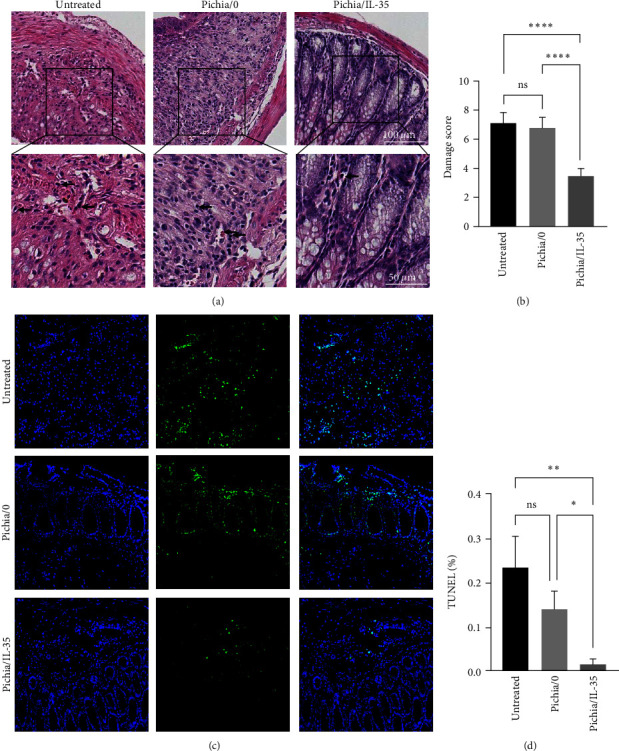
Pichia/IL-35 alleviated colon histological damage and apoptosis in colitis mice. (a) Representative H&E-stained sections of the distal colon after DSS administration and Pichia/0 or Pichia/IL-35 treatment (magnification: 200x). (b) Histology scores of mice colon in the untreated, Pichia/0 and Pichia/IL-35 groups at day 10 after DSS administration (*n* = 6 per group). (c) TUNEL images acquired by fluorescence microscope (magnification: 200x). The green part in the picture represents apoptotic cells in colon tissue (d) Percentage of apoptotic cells. All data are presented as mean ± SD. Inside the box is a 400x magnification image. One-way ANOVA was used to perform statistical analysis. ns = no significant,  ^*∗*^*p* < 0.05,  ^*∗∗*^*p* < 0.01, and  ^*∗∗∗∗*^*p* < 0.0001.

As expected, inflammatory cell infiltration, mucosal damage, and crypt structure destruction were observed in the untreated and Pichia/0 groups. These changes were significantly attenuated and histopathological scores were significantly lower in the Pichia/IL-35 group than those of the untreated and Pichia/0 groups (Pichia/IL-35 group vs. Pichia/0 group, *p* < 0.0001; Pichia/IL-35 group vs. untreated group, *p* < 0.0001) (Figures 3(a) and 3(b)). Meanwhile, compared with the untreated group and Pichia/0 group, the apoptosis of Pichia/IL-35 group was significantly reduced (Pichia/IL-35 group vs. Pichia/0 group, *p* < 0.05; Pichia/IL-35 group vs. untreated group, *p* < 0.01) (Figures 3(c) and 3(d)).

### 3.4. Pichia/IL-35 Increased the Proportion of M2 Macrophages and Reduced the Level of Pro-Inflammatory Cytokines in DSS-Induced Colitis Mice

**Figure 4:**
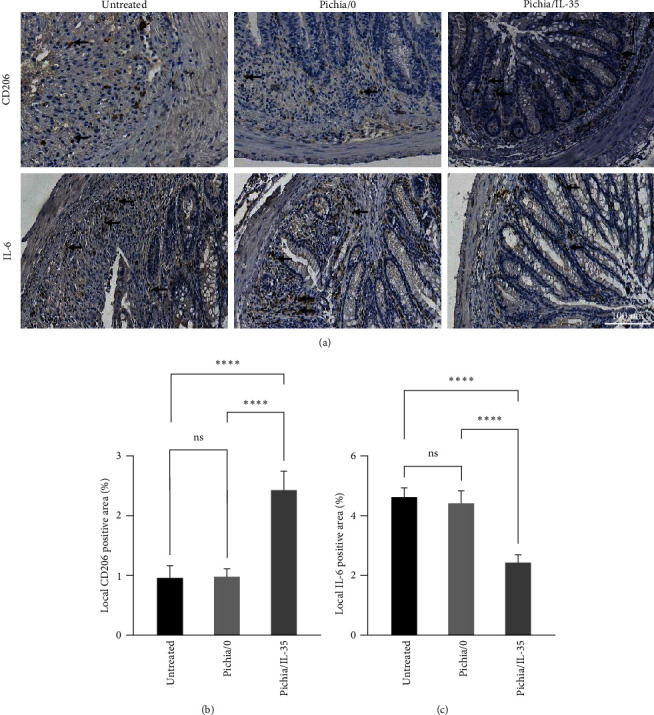
Pichia/IL-35 increased the proportion of M2 macrophages and reduced the level of pro-inflammatory cytokines in DSS-induced colitis mice. To evaluate the effect of Pichia/IL-35 on local immune microenvironment, CD206 and IL-6 (a) immunohistochemistry of colon sections in untreated, Pichia/0 and Pichia/IL-35 treated mice were performed. Quantitative data of cell positive area for each group are shown in (b) and (c) respectively (*n* = 6 per group). All data are presented as mean ± SD. The arrow shows positive cells. Statistics analyzed by One-way ANOVA. ns = no significant,  ^*∗∗∗∗*^*p* < 0.0001.

To investigate the effects of Pichia/IL-35 on the regulation of macrophages and pro-inflammatory cytokines in the colon, the infiltration of M2 macrophages (CD206^+^ cell) and level of IL-6 was examined. As shown in Figure 4, the Pichia/IL-35 treatment increased CD206^+^ cell infiltration but decreased IL-6 expression compared with those of Pichia/0 and untreated groups (CD206: Pichia/IL-35 group vs. Pichia/0 group, *p* < 0.0001; Pichia/IL-35 group vs. untreated group, *p* < 0.0001; IL-6: Pichia/IL-35 group vs. Pichia/0 group, *p* < 0.0001; Pichia/IL-35 group vs. untreated group, *p* < 0.0001). This suggests that Pichia/IL-35 can affect macrophage polarization and cytokine secretion.

### 3.5. Pichia/IL-35 Decreased the Percentages of Th1, Th17, and M1 Macrophages but Increased the Percentage of Tregs in MLNs of DSS-Induced Colitis

**Figure 5:**
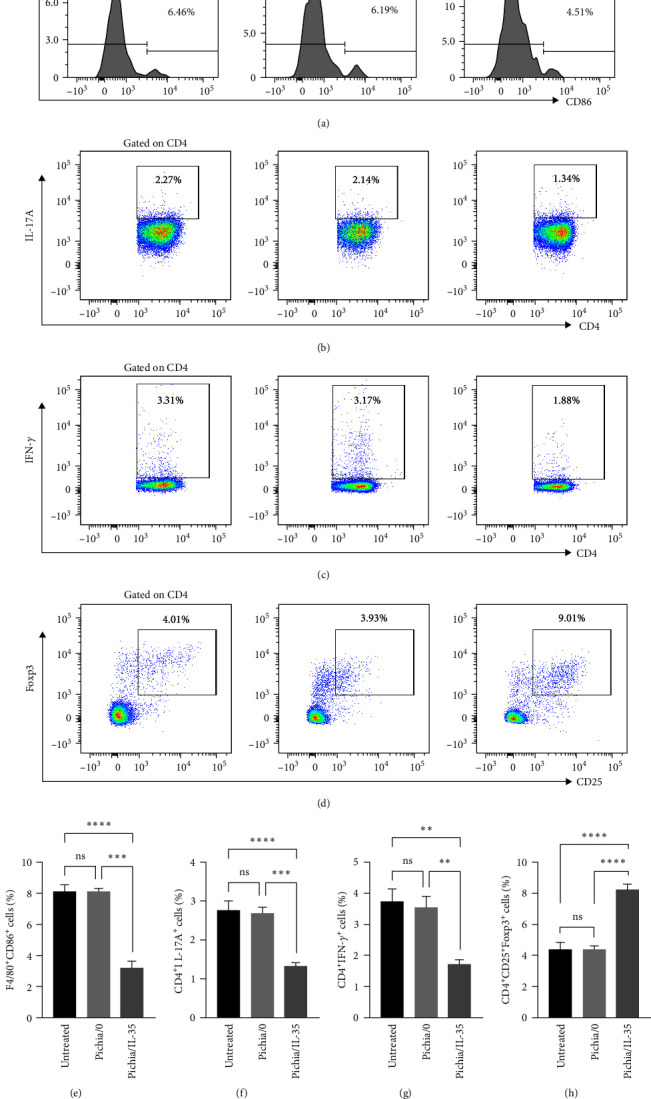
Pichia/IL-35 decreased the percentages of Th1, Th17, and M1 macrophages but increased the percentage of Tregs in MLNs of DSS-induced colitis. In addition to evaluation of the colon tissue, cell suspensions of MLNs were also prepared and stained for flow cytometry analysis. The representative histogram of (a) M1 (F4/80^+^CD86^+^) macrophages, the pseudocolor of (b) Th17 (CD4^+^IL-17A^+^), (c) Th1 (CD4^+^IFN-*γ*^+^) cells and (d) Tregs (CD4^+^CD25^+^Foxp3^+^) were depicted. In addition, the percentage of (e) M1 macrophages, (f) Th17 cells, (g) Th1 cells, (h) Tregs were calculated and graphed (*n* = 6 per group). All data are presented as mean ± SD. Statistics analyzed by One-way ANOVA. ns = no significant,  ^*∗∗*^*p* < 0.01,  ^*∗∗∗*^*p* < 0.001, and  ^*∗∗∗∗*^*p* < 0.0001.

To determine the immunomodulatory effects of Pichia/IL-35, cell suspensions of MLNs were prepared and stained for flow cytometry analysis. As shown in Figures 5(a), 5(b), and 5(c), compared with untreated and Pichia/0 groups, Pichia/IL-35 treatment could decrease the percentages of M1 macrophages, Th17 cells and Th1 cells in the MLNs (Figure 5(e), M1: Pichia/IL-35 group vs. Pichia/0 group, *p* < 0.001; Pichia/IL-35 group vs. untreated group, *p* < 0.0001; Figure 5(f), Th17: Pichia/IL-35 group vs. Pichia/0, *p* < 0.001; Pichia/IL-35 group vs. untreated group, *p* < 0.0001; Figure 5(g), Th1: Pichia/IL-35 group vs. Pichia/0, *p* < 0.01; Pichia/IL-35 group vs. untreated group, *p* < 0.01). In addition, Pichia/IL-35 treatment could increase the percentages of Tregs, which may promote immune tolerance (Pichia/IL-35 group vs. Pichia/0 group, *p* < 0.0001; Pichia/IL-35 group vs. untreated group, *p* < 0.0001) (Figures 5(d) and 5(h)). This suggests that Pichia/IL-35 successfully exerts immunomodulatory functions in mice with colitis.

### 3.6. IL-35 Promoted the Expression of TCPTP to Inhibit the Phosphorylation of STAT1

**Figure 6:**
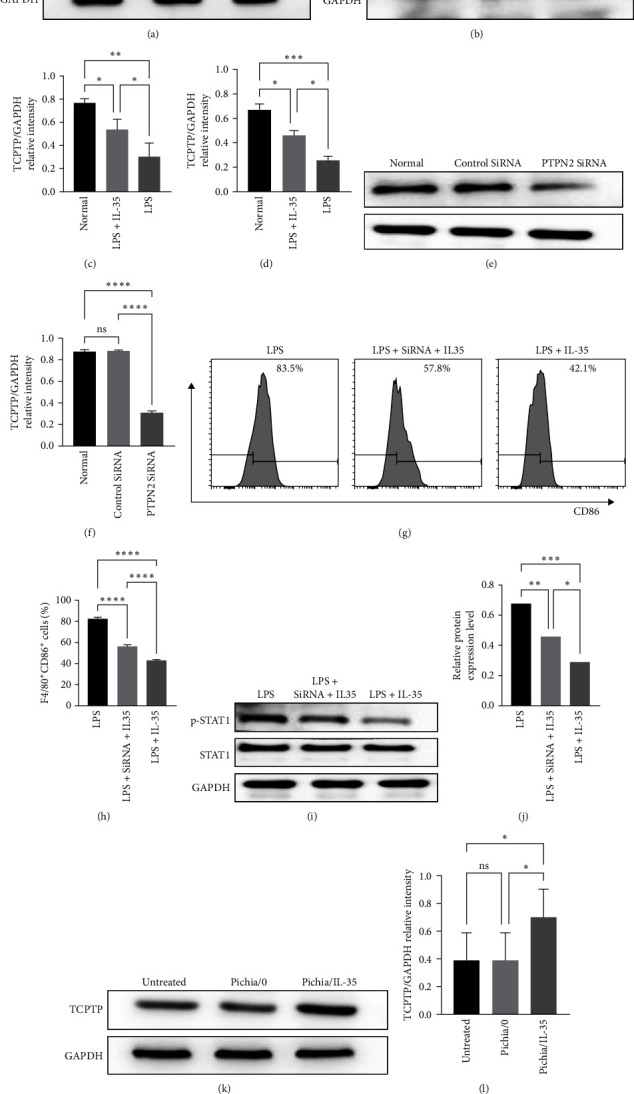
IL-35 promoted TCPTP expression *in vivo* and *in vitro*. To access the effect of IL-35 on the expression of TCPTP in macrophages *in vitro*, RAW264.7 cell line and bone-marrow derived macrophages (BMDMs) were used. Representative western blots image of the TCPTP proteins from RAW264.7 (a) and BMDMs (b) were shown. The western blots image of SiRNA knockdown was shown as (e). The percentages of M1 macrophages and the western blots image in different groups was shown as (g, i). *In vivo*, we chose to detect the expression of TCPTP in colon tissue, and the representative western blots image was shown as (k). GAPDH was used as a loading control. (c, d, f, h, j, and l) shows the statistical graphs, respectively. All data are presented as mean ± SD. Statistics analyzed by one-way ANOVA. ns = no significant,  ^*∗*^*p* < 0.05,  ^*∗∗*^*p* < 0.01,  ^*∗∗∗*^*p* < 0.001, and  ^*∗∗∗∗*^*p* < 0.0001.

TCPTP plays an anti-inflammatory role in natural and acquired immunity. In this study, the expression of TCPTP in macrophages and in intestinal tissues was examined separately to identify IL-35-mediated therapeutic efficacy. *In vitro*, IL-35 recombinant protein could promote TCPTP expression in macrophages (RAW264.7, BMDMs) compared with that of untreated group under the stimulation with LPS (Figures 6(a), 6(b), 6(c), and 6(d); RAW264.7: LPS + IL-35 group vs. LPS group, *p* < 0.05; BMDM: LPS + IL-35 group vs. LPS group, *p* < 0.05). In order to confirm that TCPTP does partially mediate the therapeutic effect of IL-35, we used siRNA to reduce the expression of TCPTP in macrophages (Figures 6(e) and 6(f), PTPN2 SiRNA group vs. control SiRNA group, *p* < 0.0001, PTPN2 SiRNA group vs. normal group, *p* < 0.0001). As expected, the percentage of M1 macrophages in LPS + SiRNA+IL-35 group was significantly higher than that of the LPS + IL-35 group (Figures 6(g) and 6(h); LPS group vs. LPS + IL-35 group, *p* < 0.0001; LPS + SiRNA+IL-35 group vs. LPS + IL-35, *p* < 0.0001). To further explore how IL-35 affects the activation of macrophage function, we detected the phosphorylation level of the STAT1, which is the substrate of TCPTP. The results showed that p-STAT1 level in the LPS + IL-35+SiRNA group was higher than that LPS + IL-35 group (Figures 6(i) and 6(j); LPS group vs. LPS + IL-35 group, *p* < 0.001; LPS + SiRNA + IL-35 group vs. LPS + IL-35, *p* < 0.05). Similarly, in the colon tissues, the expression of TCPTP was higher in the IL-35 group than that of the untreated and Pichia/0 groups (Figures 6(k) and 6(l); Pichia/IL-35 group vs. Pichia/0 group, *p* < 0.05; Pichia/IL-35 group vs. untreated group, *p* < 0.05).

## 4. Discussion

DSS-induced colitis is a general-recognized experimental model of acute IBD and is widely used to evaluate potential new therapies toward UC [53]. In the present study, a unique engineered strain that can continuously express IL-35 was successfully constructed to systematically investigate its protective effects in DSS-induced colitis. Pichia pastoris (*P. pastoris*) was used in this study for its protein expression mechanism is similar to that of mammalian cells, and its advantages in expansion speed, optimal protein modification and secretion, and easy genetic modification compared with bacteria (such as *Escherichia coli*) [22]. At the same time, compared with systemic administration, oral administration is more convenient and safer. To our delight, oral administration of Pichia/IL-35 significantly improved symptoms and reduced colonic histopathological damage scores in mice with DSS-induced colitis compared with mice in Pichia/0 and untreated groups. In addition, Pichia/IL-35 decreased the infiltration of M1 macrophages in the MLNs and the level of IL-6 in colon tissues. Besides that, upregulation of M2 macrophages and Tregs while downregulation of Th1 and Th17 cells was detected in the MLNs after Pichia/IL-35 treatment. Taken together, oral administration of Pichia/IL-35 could attenuate DSS-induced colitis.

UC is a multifactorial disease with unclear etiology, in which genetic and environmental factors are thought to be the most important risk factors [54]. Antigen recognition and dysregulation of immunological responses are essential in the progression of UC [54]. Therefore, the strategy to regulate the local immune microenvironment balance is a promising choice for UC therapy. In our study, we found that oral administration of Pichia/IL-35 could attenuate the DSS-induced colitis, which was paralleled with Zhang et al. [20]and Wang et al. [55] previous report [20, 55]. IL-35 is a newly identified member of IL-12 family, which can suppress the immunity *via* induction of Tregs and suppression of Th cells [56]. Oral administration of Pichia/IL-35 may provide an effective strategy to steadily and continuously produce IL-35 in the intestinal luminal, which could promote the foundation of immune homeostasis, and eventually protect the mice from DSS-induced colitis.

The imbalance between Th17 and Treg has been verified to participate in the initiation and progress of UC [57, 58, 59], elevated Th17 cells and decreased Tregs were observed in colon tissues of UC patients and experimental colitis model [51, 60, 61]. As an immunomodulatory cytokine, IL-35 has been confirmed to possess the capacity of promoting Treg generation and decreasing inflammatory Th17 cells both in mice and human [11, 62]. Wirtz et al. [63] revealed that the mucosa in EBI3-deficient mice with spontaneous or T-cell transfer-induced colitis infiltrated large amounts of activated CD4^+^ T cells producing Th1 and Th17 cytokines, and recombinant IL-35 treatment significantly ameliorated the colitis and decreased proportions of Th1 and Th17 cells. Our previous study reported that oral administration *E. coli*/IL-35 significantly increased the level of CD4^+^CD25^+^Foxp3^+^ Tregs while reduced the Th17 cells both in splenocytes and MLNs [20]. Similarly, here we observed the same results that Pichia/IL-35 could regulate the balance of Th17/Treg to attenuate the experimental colitis.

Apart from adaptive immunity characterized by T cells, innate immunity also plays a significant role in the initiation of UC [64]. As an important component of the innate immunity, macrophages have been identified pivotal for colitis therapy [65]. Macrophages can differentiate into M1 or M2 subtypes according to the surroundings, M1 macrophages promote inflammation, while M2 macrophages restrain inflammation [66, 67]. Hence, modulating macrophages may provide novel insights for treating colitis. For example, aminosalicylates downregulate NF-*κ*B phosphorylation, thiopurines inhibit the activity of GTPase Rac1, both of them can inhibit the activation of macrophages [68, 69], and anti-TNF-*α* antibodies can promote M2 macrophage differentiation [70]. Herein, we have detected macrophages both in the colon tissues and the MLNs, and the results showed that Pichia/IL-35 elevated the proportion of M2 macrophages while reduced the M1 subsets. Previous studies have demonstrated that TCPTP played an anti-inflammatory role both in the innate and adaptive immune system [32, 33], regulated the activation and function of macrophages through different pathways, and alleviated macrophage-associated diseases, including UC [32]. Based on the results that expression of TCPTP in macrophages was confirmed higher in the Pichia/IL-35-treated group than that of the untreated group *in vitro* and *in vivo* experiments, we speculated that Pichia/IL-35 mediated the attenuation of DSS-induced colitis in mice at least partly regulates macrophage through the upregulation of TCPTP.

However, some limitations remain in this study, for that TCPTP expression in intestinal macrophages was not detected, and the underlying mechanisms of IL-35-mediated upregulation of TCPTP in macrophages were not addressed. In the future, further in-depth exploration of the detailed mechanisms of IL-35-mediated therapeutic efficacy is warranted.

## 5. Conclusion

**Figure 7:**
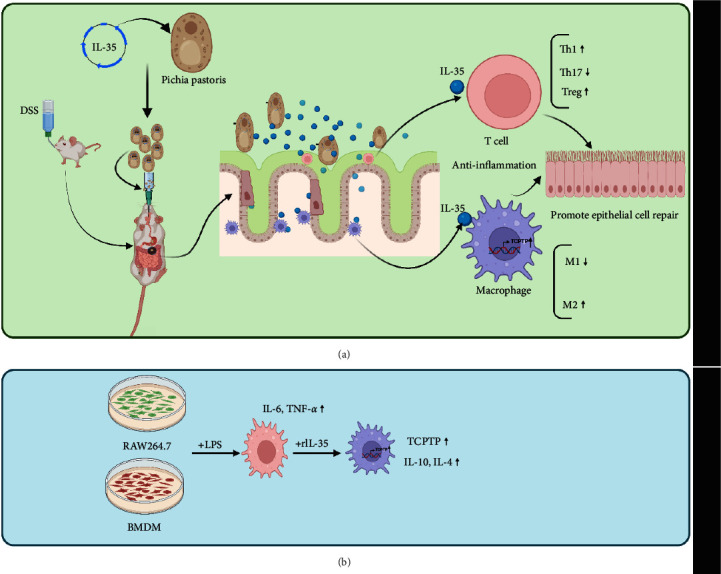
Upregulation of TCPTP in Macrophages is Involved in IL-35 Mediated Attenuation of Experimental Colitis. The Pichia pastoris expressing IL-35 was intragastrically injected into colitis mice, and the results showed that Pichia/IL-35 treatment induced T cells and macrophages to play an anti-inflammatory role in promoting intestinal epithelial repair in vivo (a). In addition, IL-35 significantly increased TCPTP expression in LPS-stimulated macrophages in vitro (b). (Created with BioRender.com).

Taken all together and as shown in Figure 7, oral administration of IL-35-producing strain Pichia/IL-35 could ameliorate experimental colitis, which may involve upregulation of TCPTP expression in intestinal macrophages.

## Data Availability

The data presented in this study are available upon request from the corresponding author or first author.
